# Establishment and characterization of a fucosylated α-fetoprotein-specific monoclonal antibody: a potential application for clinical research

**DOI:** 10.1038/s41598-019-48821-x

**Published:** 2019-08-26

**Authors:** Yuriko Egashira, Masatoshi Suganuma, Yukiko Kataoka, Yukiko Higa, Nobuyuki Ide, Koichi Morishita, Yoshihiro Kamada, Jianguo Gu, Koji Fukagawa, Eiji Miyoshi

**Affiliations:** 1 Bio-Diagnostic Reagent Technology Center, Sysmex Corporation, Takatsukadai, Nishi-ku, Kobe, 651-2271 Japan; 2 Central Research Laboratory, Sysmex Corporation, Tonomachi, Kawasaki-ku, Kawasaki, Kanagawa 210-0821 Japan; 3 Central Research Laboratory, Sysmex Corporation, Takatsukadai, Nishi-ku, Kobe, 651-2271 Japan; 4 Department of Molecular Biochemistry & Clinical Investigation, Osaka University Graduate School of Medicine, Yamada-oka, Suita, 565-0871 Japan; 5 Division of Regulatory Glycobiology, Institute of Molecular Biomembrane and Glycobiology, Tohoku Medical and Pharmaceutical University, Komatsushima, Aobaku, Sendai, 981-8558 Miyagi Japan

**Keywords:** Glycobiology, Biomaterials - proteins

## Abstract

The *Lens culinaris* agglutinin (LCA)-reactive fraction of α-fetoprotein (AFP-L3) is a well-known cancer biomarker for hepatocellular carcinoma (HCC) with very high specificity. Because LCA recognizes only bi-antennary *N*-glycans with a core fucose, some of fucosylated AFP in HCC patients may not be detected. Then glycan antibodies, which recognize both specific glycan and protein, are desired for glycobiology. Here, we successfully established a novel glycan antibody for fucosylated AFP and demonstrated its potential clinical application. After immunization with a fucosylated AFP peptide, positive screening was performed for fucosylated AFP peptides using solid-phase enzyme-linked immunosorbent assay (ELISA). The newly developed antibody was designated: fucosylated AFP-specific mAb (FasMab). Western blot analysis showed that FasMab reacted with AFP produced by HepG2 cells, but not with AFP produced by α-1,6-fucosyltransferase deficient HepG2 cells. The specific binding of FasMab to fucosylated AFP was confirmed with ELISA as well as western blot analysis. A preliminary high sensitivity chemiluminescence enzyme immunoassay kit showed increased levels of fucosylated AFP in the sera of patients with HCC, but not in the sera of normal patients, or patients with chronic liver diseases. Thus, the novel glycan antibody, FasMab, is a promising tool to study fucosylated AFP with clinical and basic research applications.

## Introduction

α-Fetoprotein (AFP) is a well-known cancer biomarker for hepatocellular carcinoma (HCC) and is used worldwide. However, the specificity of AFP for HCC diagnoses is relatively low, as it is sometimes increased in patients with chronic liver diseases, such as chronic hepatitis and liver cirrhosis. In contrast, the *Lens culinaris* agglutinin (LCA)-reactive fraction of α-fetoprotein (AFP-L3) is specifically increased in patients with HCC^1,2^. AFP has a bi-antennary complex-type structure that may be modified with a fucose residue^3^. AFP-L3 is produced by α-1,6-fucosyltransferase (FUT8), which is involved in the synthesis of core fucose modifications on *N*-glycans^4^. To understand the molecular mechanisms underlying the process by which fucosylated AFP is specifically increased in the sera of HCC patients, we previously purified the FUT8 protein and cloned the cDNA from the porcine brain and conditioned medium of a human gastric cancer cell line MKN45^4,5^. While the expression level of FUT8 in the normal liver is quite low, it increases in patients with chronic liver diseases, like liver cirrhosis, as well as in HCC patients^6^. In addition to FUT8 expression, other molecules that regulate fucosylation, such as GDP fucose and the GDP-fucose transporter, are increased in HCC cells and tissue^7,8^.

Affinity electrophoresis using LCA is widely used to measure fucosylated AFP in patient sera^9^. However, this method has several problems. First, AFP-L3 levels can be inaccurately measured by LCA affinity electrophoresis because of *N*-glycan branching^10^. Second, it is impossible to measure AFP-L3 in countries that do not have the automated immunoassay system. To overcome these problems, it is critical to develop a glycan antibody for fucosylated AFP. While other glycan antibodies recognize only oligosaccharides, such as sialyl-Lewis A or X, at non-reducing terminal regions of sugar chains^11^, novel glycan antibodies recognize both characteristic oligosaccharides and peptides, and represent bona fide tools of next-generation glycobiology. However, it is very difficult to establish this type of glycan antibody for fucosylated AFP using normal immunization methods, largely because α-1,6-fucosylation is a common glycan modification in antibody-producing animals like mice and rabbits^12,13^ and α-1,6-fucose itself has low immunogenicity. Although existing methods to establish anti-carbohydrate antibodies use immunization of glycosylation related gene-deficient mice^14,15^, it is difficult to use this model to establish an α-1,6-fucose specific antibody because *FUT8*-deficient mice have much lower survival rates than wild-type mice and severe growth retardation and immunodeficiency^16^.

In the present study, we developed a fucosylated protein-specific mAb for fucosylated AFP (designated FasMab) using glycopeptide immunization, and characterized FasMab using several types of assays, including epitope mapping. Furthermore, we investigated whether the FasMab can be used for the detection of fucosylated AFP in sera of HCC patients using a preliminary High Sensitivity Chemiluminescence Enzyme Immunoassay (HISCL) kit.

## Materials and Methods

### Glycopeptide design for the fucosylated AFP-specific mAb

**Figure 1:**
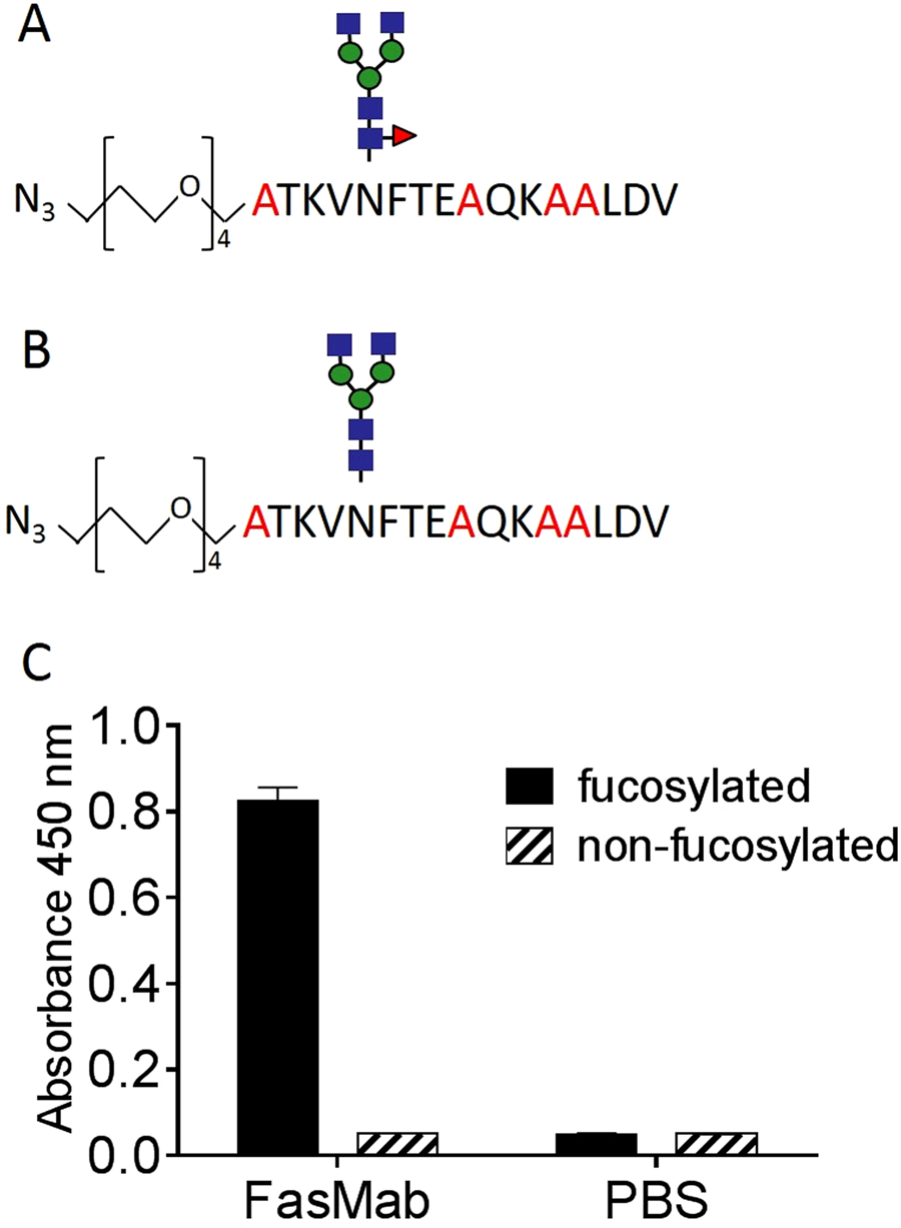
Establishment of a mAb targeting fucosylated AFP peptides. Schematic representation of the fucosylated AFP peptide (**A**) and non-fucosylated AFP peptide (**B**) used for immunization and hybridoma screening. Positions of mutated amino acids are shown in red. Blue squares, green circles, and red triangles correspond to *N*-acetylglucosamine, mannose, and fucose, respectively. (**C**) Bar graph illustrating the reactivity of the selected antibody (FasMab) with the fucosylated or non-fucosylated AFP peptides. Error bars represent the standard deviation.

To produce the fucosylated protein-specific mAb against fucosylated AFP, we used a glycopeptide design for immunization of mice, shown in Fig. 1A. AFP glyco-peptide was used for immunization, which contains 16 amino acids including Asn-232 where AFP has only one *N*-glycan^17^. To reduce the immunogenicity of epitopes other than the area around the glycosylation site, certain amino acids were substituted for alanine. We attached the following oligosaccharide: GlcNAc_2_Man_3_GlcNAc_2_Fuc, which is found in the sera of patients with HCC^18^. The fucosylated AFP peptide and non-fucosylated counterpart for the negative control (Fig. 1B) were purchased from GlyTech (Kyoto, Japan). Keyhole limpet hemocyanin (KLH) was conjugated to the fucosylated AFP peptide for immunization and bovine serum albumin (BSA) was conjugated to the fucosylated and non-fucosylated AFP peptides for hybridoma screening. These peptide conjugations were performed using click chemistry by GlyTech.

### Cell culture

The human hepatocellular carcinoma cell lines, Huh7, HepG2, and *FUT8*^−/−^ HepG2 cells^19^ were cultured in DMEM (Thermo Fisher Scientific, Waltham, MA, USA) containing 10% fetal bovine serum (GE Healthcare, Buckinghamshire, UK) with or without 100 units/mL penicillin and 100 μg/mL streptomycin at 37 °C in 5% CO_2_ humidified conditions.

### Preparation of afucosylated AFP

AFP purified from human cord serum (Lee Biosolutions, Maryland Heights, MO, USA) was diluted with buffer A (50 mM Tris-H_2_SO_4_, pH 7.3) and the solution was subjected to chromatography on a LCA-high-performance liquid chromatography (HPLC) column (J-OIL MILLS, Tokyo, Japan) using the same buffer. The flow-through fraction was collected and dialyzed with phosphate-buffered saline (PBS) and the AFP concentration was determined by measuring absorbance at 280 nm using a Nanodrop spectrophotometer (Thermo Fisher Scientific).

### Preparation of fucosylated AFP

For the purification of AFP, a goat anti-AFP polyclonal antibody (LifeSpan BioSciences, Seattle, WA, USA) was conjugated to a HiTrap NHS-activated HP column (GE Healthcare) according to the manufacturer’s instructions. Huh7 cells were cultured in serum-free DMEM for 6 days and conditioned medium was collected at the end of the 6 days. Proteins from Huh7 conditioned media were precipitated using 70% saturated ammonium sulfate, and the precipitate was resuspended in 20 mM phosphate (pH 7.0) and 150 mM NaCl; the sample was then applied to an anti-AFP antibody-coupled column equilibrated with the same buffer. Proteins bound to the column were eluted with 100 mM Glycin-HCl (pH 2.7) and 150 mM NaCl. The purified AFP solution was dialyzed in buffer A and the solution was subjected to chromatography on an LCA-HPLC column (J-OIL MILLS). Fucosylated proteins were eluted with buffer A containing 400 mM methyl-D-glucoside and were dialyzed in PBS. The protein concentration was determined by measuring absorbance of the solution at 280 nm using a Nanodrop spectrophotometer (Thermo Fisher Scientific).

### Preparation of AFP from HepG2 cells

The conditioned media of wild-type or *FUT8*^−/−^ HepG2 cells was diluted with 20 mM Phosphate (pH 7.0) and 150 mM NaCl and applied to an anti-AFP antibody-coupled column. Bound proteins were eluted with 100 mM Glycin-HCl (pH 2.7) and 150 mM NaCl, then dialyzed in PBS. Protein concentration was determined by measuring the absorbance of the solution at 280 nm using a Nanodrop spectrophotometer (Thermo Fisher Scientific).

### Glycan analysis

Proteins were deglycosylated by a PNGase F (Takara Bio, Shiga, Japan) treatment. The released *N*-glycans were purified and labeled with 2-aminopyridine using BlotGlyco (Sumitomo Bakelite, Tokyo, Japan) according to the manufacturer’s instructions. Liquid chromatography-mass spectrometry (LC-MS) analysis was performed by Sumitomo Bakelite, using LC-MS-IT-TOF (Simadzu, Kyoto, Japan). *N*-glycan structures were predicted using the GlycoMod Tool (http://web.expasy.org/glycomod/).

### Experimental animals

BALB/cA mice and New Zealand White rabbits were used to obtain antibodies. The mice and rabbits were treated in accordance with the guidelines and regulations related to animal research, including Act on Welfare and Management of Animals, Standards relating to the Care and Keeping and Reducing Pain of Laboratory Animals and Standards relating to the Methods of Destruction of Animals. The protocols for the experiment animals were approved by the animal welfare review committee of BEX (Tokyo, Japan) or KITAYAMA LABES (Nagano, Japan).

### Hybridoma production of the fucosylated AFP-specific mAb

The fucosylated protein-specific mAb against fucosylated AFP (FasMab) was developed by immunizing BALB/cA mice with a KLH conjugated fucosylated AFP peptide (GlyTech). Splenocytes from immunized mice were fused with P3U1 myeloma cells to generate hybridoma cell lines according to a standard procedure. Antibodies specific to the fucosylated AFP peptide were screened by ELISA using BSA-conjugated fucosylated or non-fucosylated AFP peptides. Two microliters/milliliter of BSA-conjugated glycopeptides, diluted in PBS, were immobilized on 96-well plates at 4 °C overnight. The wells were washed with PBS + 0.05% Tween-20 (PBS-T) and blocked with 0.5% skim milk in PBS at room temperature (RT) for 30 min. The plates were then incubated with hybridoma culture supernatant at RT for 1 h, followed by addition of alkaline phosphatase (ALP)-labeled goat anti-mouse IgG (SouthernBiotech, Birmingham, AL, USA). The enzymatic reaction was conducted using p-nitrophenyl phosphate. Optical density was measured at 492 nm using an MTP-310 microplate reader (CORONA ELECTRIC, Ibaraki, Japan).

### Hybridoma production of in-house developed mouse anti-AFP mAb

The mouse anti-AFP mAb Clone 2B12 we developed in-house for sandwich ELISA with FasMab was obtained by immunizing BALB/cA mice with AFP protein from human cord serum (Lee Biosolutions). Splenocytes from immunized mice were fused with P3U1 myeloma cells to generate hybridoma cell lines using the same method as that used to develop FasMab. Antibodies specific to AFP protein were screened using an ELISA assay with the AFP protein. Two micrograms/milliliter of AFP protein diluted in PBS was immobilized on 96-well plates at 4 °C overnight. The wells were washed with PBS-T and blocked with 0.5% skim milk in PBS at RT for 30 min. Then, plates were incubated with hybridoma culture supernatant at RT for 1 h, followed by addition of ALP-labeled goat anti-mouse IgG (SouthernBiotech). The enzymatic reaction was conducted using p-nitrophenyl phosphate. Optical density was measured at 492 nm using an MTP-310 microplate reader (CORONA ELECTRIC).

### In-house developed rabbit anti-AFP mAb production

In order to construct a highly sensitive sandwich ELISA with FasMab, an anti-AFP antibody was developed using a rabbit antibody phage display system. Rabbit antibody cDNAs were derived from the spleen cells of two New Zealand White rabbits immunized with AFP protein from human cord serum (Lee Biosolutions). Rabbit antigen binding fragment (Fab) libraries in the phagemid vector were generated from the cDNAs as described^20,21^. Phage clones specific to AFP were obtained through two round panning using AFP-immobilized plates. Phage ELISA was performed, using an ELISA plate coated with 200 ng AFP protein or 500 ng mouse IgG (as a negative control). *E. coli* culture supernatants or purified phages diluted with 2% skim milk were added to the plates and incubated at RT for 1 h. After the plates were washed with PBS-T, an HRP-conjugated anti-M13 antibody (GE Healthcare) was added to the plates and incubated at RT for 1 h. The enzymatic reaction was conducted using 3,3′,5,5′-tetramethylbenzidine (TMB) substrate and optical density was measured at 450 nm using a SpectraMax 190 Microplate Reader (Molecular Devices). The positive clones were subjected to sequence analysis.

### Molecular cloning of FasMab

Total RNA was extracted from the established hybridoma cells producing FasMab by using ISOGENII (NIPPON GENE, Toyama, Japan). Next, the cDNA was synthesized by reverse transcription PCR and the antibody nucleotide sequences were amplified using mouse IgG specific primers and the SMARTer RACE 5′/3′ kit (Takara Bio). The PCR products were cloned into the pMD20 vector (Takara Bio) and the nucleotide sequences were confirmed using M13 universal primers.

### Expression and purification of mAbs developed in this study

The heavy chain and light chain genes of FasMab and the rabbit anti-AFP mAb were subcloned into the pcDNA3.4 vector (Thermo Fisher Scientific). These mAbs were expressed in Expi293 cells (Thermo Fisher Scientific) according to the manufacturer’s instructions, and purified from clarified and filtered Expi293 cells culture supernatant using MabSelect SuRe (GE Healthcare). The in-house developed mouse anti-AFP mAb Clone 2B12 was purified from conditioned media of the hybridoma. Concentrations of all three mAbs were determined by measuring absorbance at 280 nm using a Nanodrop spectrophotometer (Thermo Fisher Scientific).

### Western blot, and lectin blot analyses

Twenty nanograms of fucosylated AFP, afucosylated AFP, ALP from calf intestine (Oriental Yeast, Tokyo, Japan) and AFP produced by wild-type or *FUT8*-deficient HepG2 cells^19^ were electrophoresed on 10–20% Tris-glycine gradient gels (ATTO, Tokyo, Japan) and transferred onto PVDF membranes (Thermo Fisher Scientific). All samples were purified proteins and ALP containing fucosylated *N*-glycans was used as a negative control. After blocking with PVDF Blocking Reagent for Can Get Signal (NOF, Tokyo, Japan), the membrane was incubated with FasMab, mouse anti-AFP mAb Clone 2B12, or the anti-ALP mAb (Rockland Immunochemicals). The membrane was washed with TBS containing 0.05% Tween-20 (TBS-T) and then incubated with peroxidase-conjugated secondary antibodies. After washing with TBS-T, the proteins were detected with an ECL kit (GE Healthcare).

The conditioned media of wild-type and *FUT8*-deficient HepG2 cells were condensed and protein concentration was quantified by using a bicinchoninic acid (BCA) protein assay kit (Nacalai tesque, Kyoto, Japan). Twenty micrograms of total protein included there was subjected to 10% SDS-polyacrylamide gel electrophoresis, and then transferred to a PVDF membrane (Millipore, Billerica, MA). After blocking with PBS containing 3% BSA or 5% skim milk, the membrane was incubated with FasMab, mouse anti-AFP mAb Clone 2B12 or biotinylated *Aleuria aurantia* lectin (AAL; J-Oil Mills). AAL was used for detection of fucosylated proteins contained in the conditioned media. AAL recognizes all types of fucosylation, that is, α1,2-, α1,3-, α1,4, and α1,6-fucose-containing oligosaccharides^22^ but it is reported that it preferentially binds to α1,6-fucosylated oligosaccharides^23^. The membrane was washed with TBS-T and then incubated with peroxidase-conjugated anti-mouse IgG for western blotting and incubated with avidin-peroxidase conjugates (ABC kit, Vector Laboratories, Burlingame, CA) for AAL blotting. After washing with TBS-T, the protein bands were detected with an ECL kit (Wako, Osaka, Japan).

### Human serum samples

In this study, 25 normal human serum samples were purchased from ProMedDx (Norton, MA, USA), 20 liver disease serum samples were purchased from BioreclamationIVT (Westbury, NY, USA), and 55 AFP-positive HCC serum samples were purchased from Access Biologicals (Vista, CA, USA) and BioreclamationIVT (Westbury, NY, USA). The serum samples purchased from Access Biologicals, ProMedDx and BioreclamationIVT have been collected through obtaining informed consent from each donor and under protocols approved by Independent IRB (Sunrise, FL, USA), New England Independent Review Board (Needham, MA, USA) and Schulman Associates IRB (Cincinnati, OH, USA) respectively. All experimental protocols were approved by the research ethics review committee of Sysmex Corporation in accordance with Ethics Guideline for Clinical Research. The AFP concentration and AFP-L3% of human serum samples were measured using µTAS WAKO AFP-L3^9^. The AFP-L3 concentration was determined using the following equation:

$${\rm{t}}{\rm{o}}{\rm{t}}{\rm{a}}{\rm{l}}\,{\rm{A}}{\rm{F}}{\rm{P}}\,{\rm{c}}{\rm{o}}{\rm{n}}{\rm{c}}{\rm{e}}{\rm{n}}{\rm{t}}{\rm{r}}{\rm{a}}{\rm{t}}{\rm{i}}{\rm{o}}{\rm{n}}\times {\rm{A}}{\rm{F}}{\rm{P}}-{\rm{L}}3{\rm{ \% }}.$$

### Immunoprecipitation and western blot analysis

For immunoprecipitation experiments, 25 μL of protein G sepharose FF (GE Healthcare) was incubated with 10 μg of a goat anti-AFP polyclonal antibody (LifeSpan Biosciences) at RT for 1 h. The resin was washed three times with PBS, and incubated with 200 μL of human serum at 4 °C overnight. After washing five times with PBS, bound proteins were eluted with 50 μL of 0.1 M glycine-HCl (pH 3.0). The solution was neutralized with 5 μL of 1 M Tris-HCl (pH 9.0). Ten microliters of the solution was electrophoresed on 10–20% Tris-glycine gradient gels (ATTO) and transferred onto PVDF membrane (Thermo Fisher Scientific). Western blot procedures were performed as purified proteins described above. Primary antibodies used included FasMab and 2B12. The band intensities were analyzed with ImageJ^24^.

### ELISA

**Figure 2:**
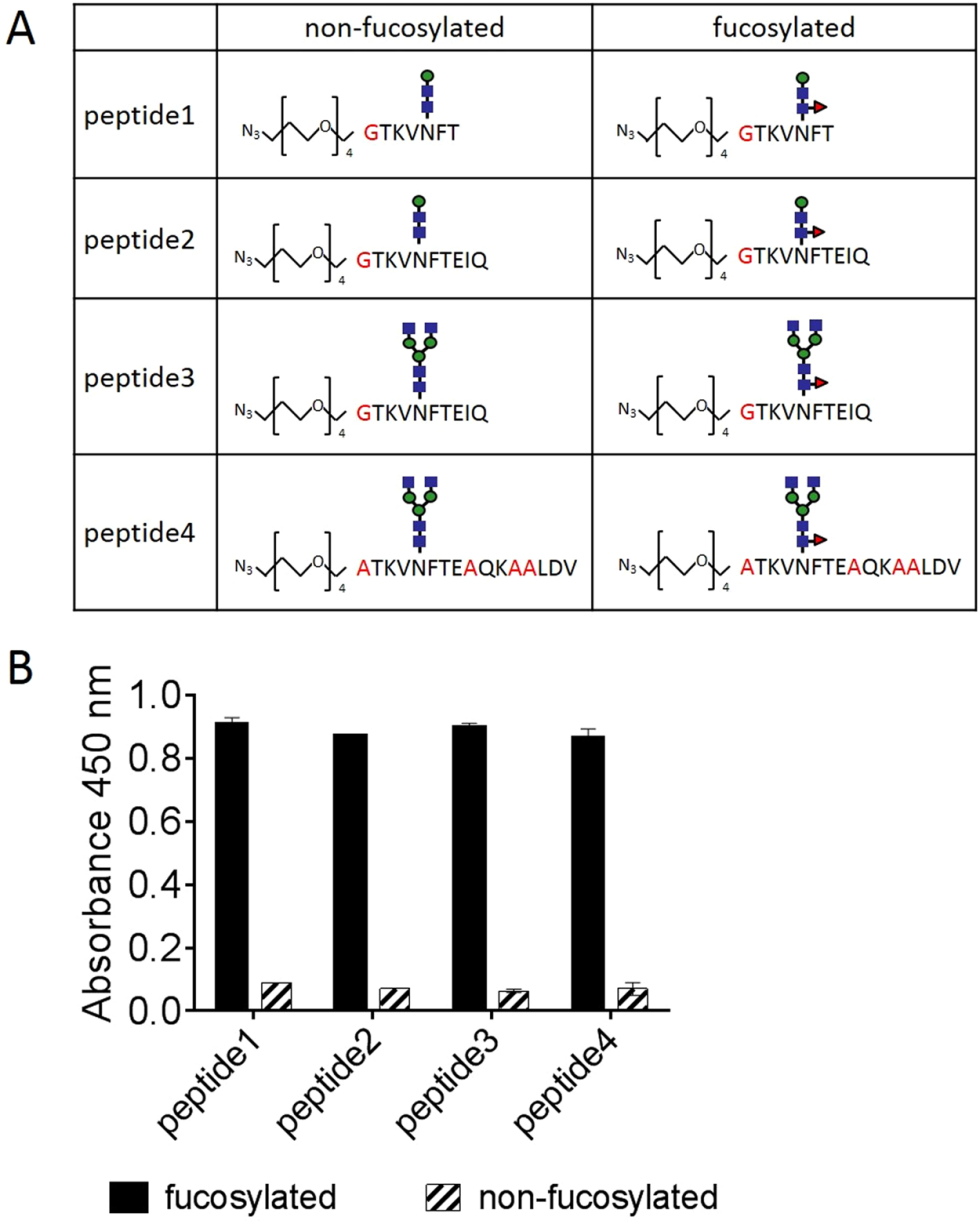
Epitope mapping of FasMab using ELISA. (**A**) Schematic representation of AFP glycopeptides. Positions of mutated amino acids are shown in red. Blue squares, green circles, and red triangles correspond to *N*-acetylglucosamine, mannose, and fucose, respectively. (**B**) Bar graph illustrating the reactivity of FasMab to the different AFP glycopeptides illustrated in (**A**). Error bars represent the standard deviation.

The glycopeptide-binding activity of the FasMab was assessed using antigen-immobilized ELISA. Two micrograms per milliliter of BSA-conjugated fucosylated or non-fucosylated AFP peptides (Fig. 2) diluted in phosphate buffer (pH 7.5) was coated onto 96-well plates at 4 °C overnight. The wells were washed with PBS-T and blocked with 1% BSA in PBS at RT for 3 h. The plates were then washed and incubated with 125 ng/mL of FasMab diluted in 1% BSA in PBS at RT for 1 h. The wells incubated with FasMab were washed, followed by addition of goat F(ab’)_2_ fragment anti-mouse IgG (H + L)-peroxidase (Beckman Coulter, Brea, CA, USA). The enzymatic reaction was conducted using TMB substrate. Optical density was measured at 450 nm using an iMark Microplate Absorbance Reader (Bio-Rad). The measurements were performed in triplicate. All data was plotted using GraphPad Prism 6 software.

The binding activity of FasMab for entire AFP protein was assessed using sandwich ELISA. FasMab (5 μg/mL) diluted in phosphate buffer (pH 7.5) was coated onto 96-well plates at 4 °C overnight. The wells were washed with PBS-T and blocked with 1% BSA in PBS at RT for 3 h. Then, the plates were incubated with fucosylated AFP, afucosylated AFP, AFP from wild-type or *FUT8*^−/−^ HepG2 cells diluted in 100 mM 2-Morpholinoethanesulfonic acid (MES) (pH 6.0) and 4 mM Tris (2-calboxyetyl) phosphine chloride (TCEP) at RT for 1 h. The wells were washed and ALP-conjugated 2B12 was added. ALP conjugation to 2B12 was performed using an Alkaline Phosphatase Labeling Kit-SH (DOJINDO, Kumamoto, Japan). After incubation at RT for 1 h, the wells were washed, and ALP substrate was added to the wells. Optical density was measured at 595 nm using a SpectraMax 190 Microplate Reader (Molecular Devices, San Jose, CA, USA). The measurements were performed in triplicate. All data was plotted using GraphPad Prism 6 software.

### Surface plasmon resonance (SPR)

Fab fragments of FasMab were prepared using immobilized Papain (Thermo Fisher Scientific) according to the manufacturer’s instructions. The interaction between fucosylated AFP and the Fab fragment of FasMab was analyzed by SPR using a Biacore X100 instrument (GE Healthcare). Fucosylated AFP was immobilized onto the surface of a CM5 sensor chip (GE Healthcare) using a standard amine coupling procedure. The kinetic data of the binding of the Fab of FasMab to antigen were obtained by injecting increasing concentrations of antibody into the sensor chip at a flow rate of 30 μL/min. Contact and dissociation time were 180 sec and 600 sec, respectively. The measurements were carried out at 25 °C in 50 mM MES (pH 6.0) 50 mM NaCl, 4 mM TCEP, and 0.005% Tween-20. Data analysis was performed with the Biacore X100 Evaluation software. Association (*k*_on_) and dissociation (*k*_off_) rate constants were calculated by a global fitting analysis and stoichiometry of (1:1). The dissociation constant (K_D_) was determined from the ratio of the kinetic rate constants as follows: K_D_ = *k*_off_/*k*_on_.

### Sandwich immunoassay for measurement of fucosylated AFP on an automated chemiluminescence enzyme immunoassay analyzer

M-280 Streptavidin Dynabeads (Thermo Fisher Scientific) were incubated with 10 µg of the biotinylated Fab fragment of FasMab in 100 mM MES (pH 6.0) at RT for 30 min. After coupling, the mAb-conjugated beads were blocked with Protein Free Blocking (Thermo Fisher Scientific). Serum sample measurements were performed in an automated chemiluminescence enzyme immunoassay analyzer (HISCL-800, Sysmex, Hyogo, Japan). Ten microliters of serum were mixed with 30 µL of 100 mM MES (pH 6.0), 16.6 mM TCEP, and 0.5% Tween-20 in a reaction tube and incubated at 42 °C for 2 min. Then, a solution containing 30 µL of 0.5% mAb-coated beads in Protein Free Blocking, 50 µg/mL Scavenger Alkaline Phosphatase (Oriental Yeast), and 0.05% Tween-20 was added to the serum sample mixture and incubated at 42 °C for 1 min. After Bound/Free separation, 100 µL of ALP-labeled Fab fragment of the in-house developed rabbit anti-AFP mAb in 120 mM MES (pH 6.5), 180 mM NaCl, 1.2 mM MgCl_2_, 0.12 mM ZnCl_2_, 1% BSA, 50 μg/mL scavenger ALP, and 0.05% Tween-20 was added to the reaction tube and incubated at 42 °C for 2.5 min. After Bound/Free separation, the ALP-conjugated anti-AFP mAb was detected using the HISCL Substrate Reagent set (Sysmex).

### Statistical analysis

Statistical analyses were performed using JMP Pro 13.0 software (SAS Institute Inc., Cary, NC). These included descriptive statistics, analysis of variance, Wilcoxon and Kruskal-Wallis tests, and Spearman’s rank correlation analysis. Differences were considered to be statistically significant at a *P* < 0.05.

## Results

### Establishment of a fucosylated AFP-specific mAb

Mice were immunized with a fucosylated AFP peptide (Fig. 1A), and harvested spleen cells were fused with P3U1 mouse myeloma cells. The conditioned medium from the resultant hybridoma was screened based on reactivity for two kinds of glycopeptides using ELISA. Positive screening was performed for the fucosylated AFP peptides (Fig. 1A) and negative screening was performed for the non-fucosylated counterpart (Fig. 1B). Among 45 positive wells, seven wells reacted to fucosylated AFP protein, but not afucosylated AFP protein, determined using western blot analysis (data not shown). Using a limiting dilution strategy, we performed single-cell cloning of the cells in each of the seven fucosylated AFP-positive wells, and obtained five hybridomas that produced antibodies that reacted to the fucosylated AFP peptide. We selected one clone, designated the fucosylated AFP specific mAb (FasMab), that had the highest binding activity to the fucosylated AFP peptide in the above-described screening. Recombinant FasMab was produced using an Expi293 cell expression system. After purification using protein affinity chromatography, the antigen-binding activity was evaluated. As shown in Fig. 1C, the recombinant FasMab bound to fucosylated AFP peptide with very high specificity.

### Epitope mapping of FasMab

We performed epitope mapping of FasMab using glycopeptide-coated ELISA, and evaluated the reactivity of FasMab to multiple AFP glycopeptides illustrated in Fig. 2A. FasMab reacted to the all fucosylated AFP peptides that contained various sugar chains/amino acids, but did not react to the non-fucosylated AFP peptides (Fig. 2B). The reactivity of FasMab with each of the four fucosylated AFP peptides was very similar, indicating that the peptide sequence after Glu-235 and the glycan residue in the non-reducing terminal of *N*-glycan has little interaction with FasMab.

### Glycan analysis

**Figure 3:**
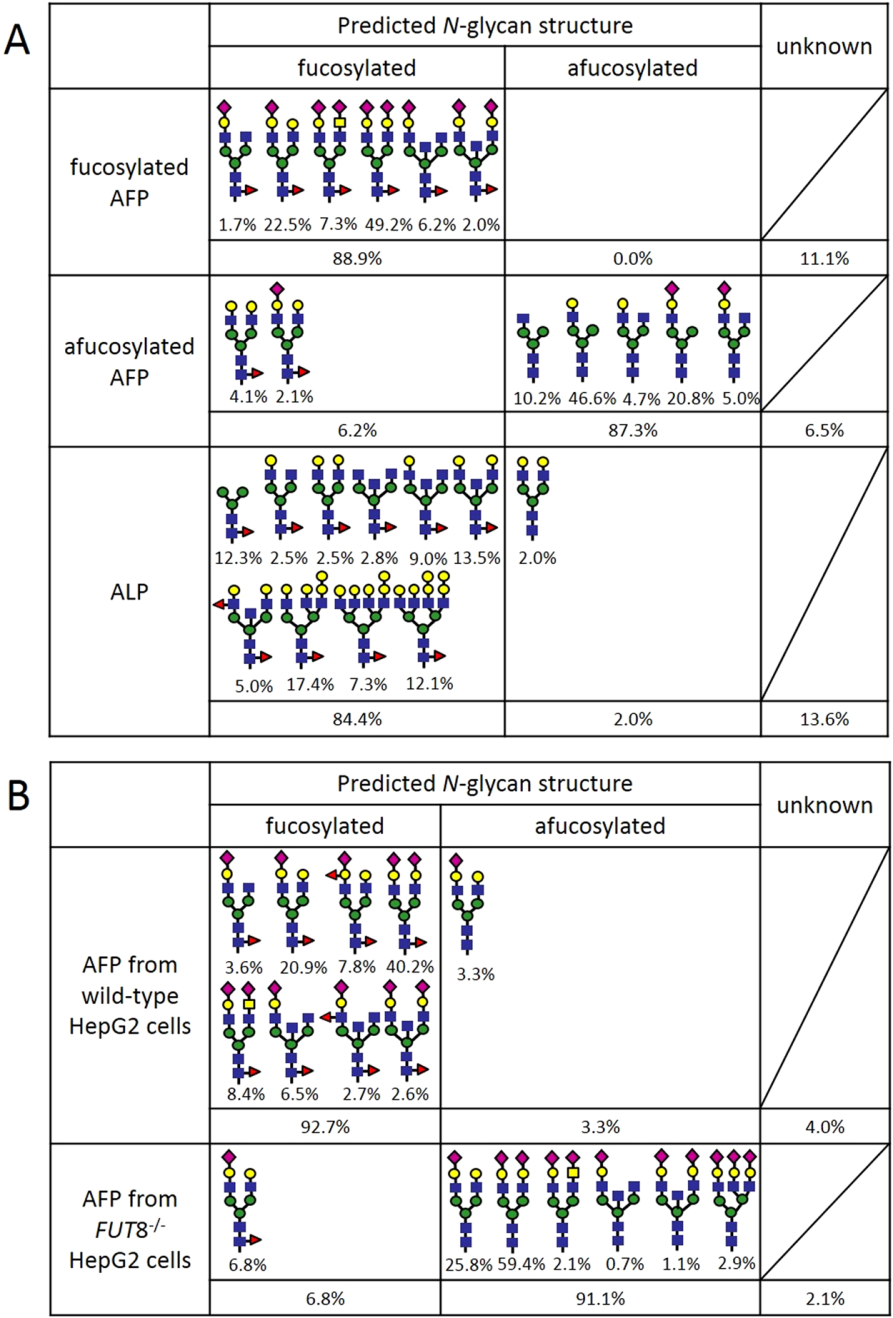
Schematic representation of the glycoforms in the proteins used in this study. (**A**) Schematic representation of the glycoforms present in fucosylated AFP, afucosylated AFP, and ALP. (**B**) Schematic representation of the glycoforms present in AFP produced by wild-type and *FUT8*^−/−^ HepG2 cells. Blue squares, green circles, yellow circles, yellow squares, magenta diamonds, and red triangles correspond to *N*-acetylglucosamine, mannose, galactose, *N*-acetylgalactosamine, sialic acid, and fucose, respectively.

To evaluate the specificity of FasMab, we prepared fucosylated AFP, afucosylated AFP, and AFP from wild-type or *FUT8*-deficient HepG2 cells with the methods described in the materials and methods section, and analyzed the *N*-glycosylation pattern of these AFPs. In addition to *N*-glycosylation patterns of the AFPs, that of commercial ALP from calf intestine, which is known to have *N*-glycans with core fucose^25^, was analyzed to examine whether FasMab shows binding activity only to fucose or fucose and specific peptide (AFP peptides). The glycosylation pattern of each protein was determined using LC-MS (Fig. 3). We found that most of the fucosylated AFP and the commercial ALP had oligosaccharide structures with fucosylated *N*-glycans, while the sialylation and branching patterns between each protein were different (Fig. 3A). The percentages of fucosylated *N*-glycans in the AFP and the ALP were 88.9% and 84.4%, respectively. In contrast, afucosylated AFP contained mainly afucosylated *N*-glycans (87.3%) and had few fucosylated *N*-glycans (6.2%). The glycosylation patterns of AFP produced by wild-type and *FUT8*^−/−^ HepG2 cells are shown in Fig. 3B. Although 92.7% of the AFP *N*-glycans produced by wild-type HepG2 cells were fucosylated, only 6.8% of the AFP *N*-glycans produced by *FUT8*^−/−^ HepG2 cells were fucosylated. It was notable that core fucosylation was still detected to a lesser extent in *N*-glycans on AFP obtained from *FUT8*^−/−^ cells; this could be due to incomplete sorting of *FUT8*^−/−^ cells from wild-type cells (performed using PhoSL lectin in FACSAria II system as previously described)^19^.

### Characterization of FasMab

**Figure 4:**
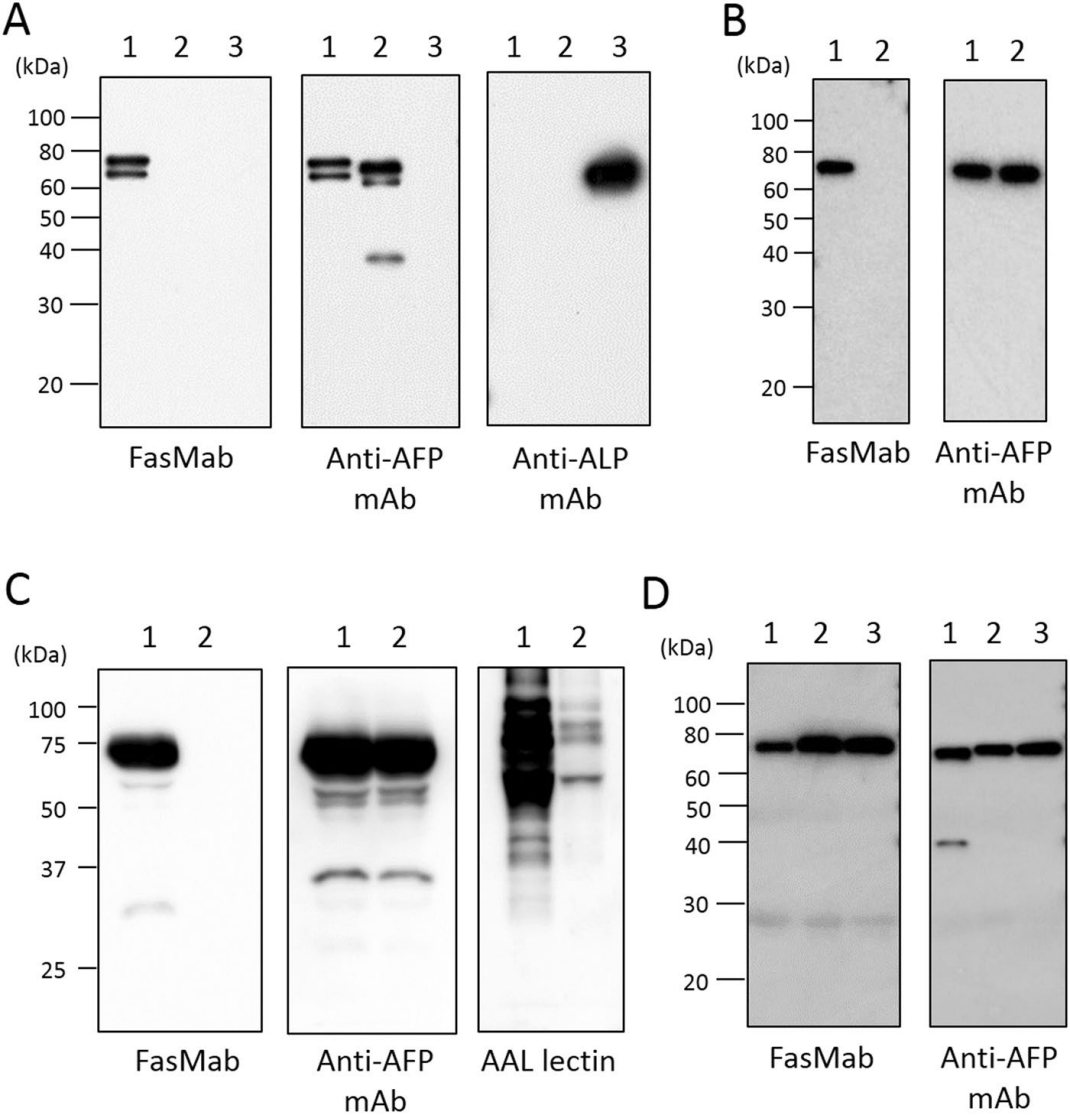
Western blot analyses. (**A**) Western blot analyses for purified fucosylated AFP, afucosylated AFP, and ALP. Lane 1: fucosylated AFP, Lane 2: afucosylated AFP, Lane 3: ALP. (**B**) Western blot analyses for AFP purified from conditioned media of wild-type and *FUT8*-deficient HepG2 cells. Lane 1: AFP from wild-type HepG2 cells, Lane 2: AFP from *FUT8*-deficient HepG2 cells. (**C**) Western blot and AAL blot analyses for conditioned media of wild-type and *FUT8*-deficient HepG2 cells. Lane 1: Supernatant of wild-type HepG2 cells, Lane 2: Supernatant of *FUT8*-deficient HepG2 cells. (**D**) Western blot analyses for AFP following co-precipitation with an anti-AFP polyclonal antibody from human serum. Lane 1: sample 1, Lane 2: sample 2, Lane 3: sample 3. All images are detected individually, and cropped from different blots. Full-length blots of (**A–D**) are presented in Supplementary Figs 1–4, respectively.

To characterize the specificity of FasMab, western blot analyses were performed (Fig. 4). First, we examined the reactivity of FasMab for fucosylated AFP, afucosylated AFP, and commercial ALP. As shown in Fig. 4A, the mouse anti-AFP mAb Clone 2B12 developed in-house reacted with both fucosylated AFP and afucosylated AFP. In contrast, FasMab reacted with fucosylated AFP, but not with afucosylated AFP or ALP. These results suggest that FasMab recognizes both fucosylated *N*-glycans and an AFP-specific peptide.

Next, we examined whether FasMab reacted with purified AFP from wild-type or *FUT8*-deficient HepG2 cells^19^. As shown in Fig. 4B, FasMab reacted with AFP produced by wild-type HepG2 cells, but not with AFP produced by *FUT8*-deficient HepG2 cells. Glycan analysis (Fig. 3) showed that AFP produced by *FUT8*-deficient HepG2 cells had few fucosylated glycans, while AFP produced by wild-type HepG2 cells was heavily fucosylated. In addition to purified AFPs, we also examined the reactivity of FasMab for AFP contained in the conditioned media of these cells. In order to determine the levels of a variety of fucosylated proteins in the conditioned media of HepG2 cells, we performed lectin blot analysis, using AAL, which is a fucose-specific lectin and preferentially binds to 1,6-fucosylated oligosaccharides fucosylated glycans^23^. As shown in Fig. 4C, FasMab recognized AFP produced by wild-type HepG2 cells, but not AFP produced by *FUT8*-deficient HepG2 cells. AAL blot analyses (Fig. 4C) show that the conditioned media of wild-type HepG2 cells contained many kinds of fucosylated proteins. These results strongly suggest that the FasMab detects the fucosylated *N*-glycans only on AFP.

We next examined whether FasMab reacted with fucosylated AFP from sera of patients with HCC. The AFP concentration and percent of AFP-L3 in the sera from three HCC patients were measured using μTAS WAKO AFP-L3. As shown in Table 1, AFP immunoprecipitated from the sera of HCC patients using a commercial anti-AFP polyclonal antibody was subjected to western blot analysis with FasMab and 2B12. While the reactivity of 2B12 was highly correlated with AFP concentrations, the reactivity of FasMab was markedly correlated with AFP-L3 concentrations (Fig. 4D, Table 1). Both FasMab and 2B12 showed no reactivity to normal human serum samples in this experiment (data not shown).

**Table 1:** Clinical background of samples and the reactivity of FasMab to the samples.

Sample	AFP (ng/mL)	AFP-L3 (%)	AFP-L3 (ng/mL)	Band intensity detected by anti-AFP mAb (arbitrary unit)	Band intensity detected by FasMab (arbitrary unit)
Sample 1	3800	27.9	1060.2	19198	17592
Sample 2	3640	88.3	3214.1	15973	30379
Sample 3	3550	93.5	3319.3	19166	29992

**Figure 5:**
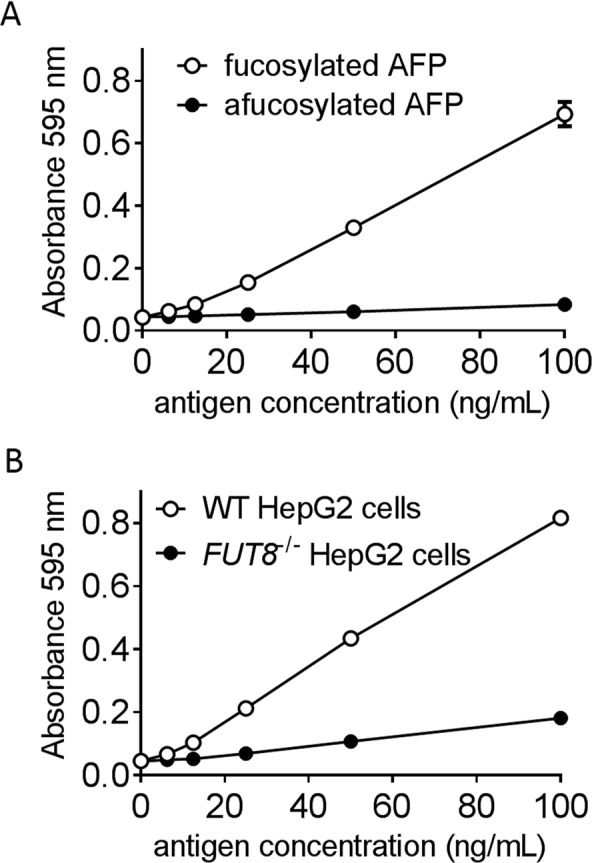
Sandwich ELISA for measuring fucosylated AFP. (**A**) Concentration-response curves for purified fucosylated AFP and afucosylated AFP. (**B**) Concentration-response curves for AFP purified from conditioned media of wild-type and *FUT8*^−/−^ HepG2 cells. Error bars represent the standard deviation.

Finally, we assessed the binding activity of FasMab to fucosylated and afucosylated AFP using sandwich ELISA. FasMab was immobilized on 96-well plates and AFP bound to FasMab was detected using ALP-labeled 2B12. As shown in Fig. 5A, fucosylated AFP bound to FasMab in a dose-dependent manner, while afucosylated AFP rarely bound to FasMab. In addition, as shown in Fig. 5B, FasMab showed much stronger binding activity for AFP produced by wild-type HepG2 cells than for AFP produced by *FUT8*^−/−^ HepG2 cells.

**Figure 6:**
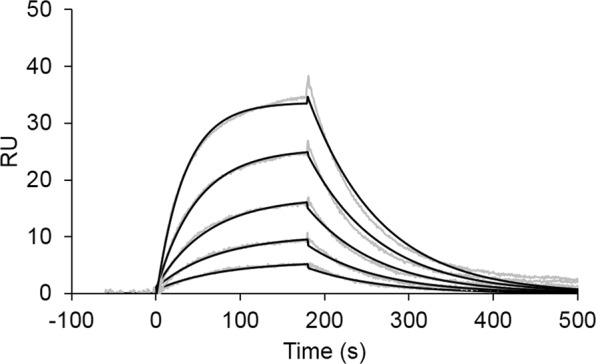
Kinetic analysis of the interaction between fucosylated AFP and FasMab. SPR sensorgrams obtained using various analyte concentrations are shown as gray lines. FasMab was used as analyte. The global fitting kinetic analyses are shown as black lines. The concentrations of the Fab fragment of FasMab analyzed were: 62.5, 125, 250, 500, and 1000 nM.

Because the affinities of most antibodies against carbohydrates are 10^3^–10^5^ times lower than antibodies against proteins and peptides^26–28^, we determined the affinity of the Fab of FasMab for fucosylated AFP using SPR (Fig. 6). The sensorgrams revealed that Fab of FasMab had a *k*_on_ rate of 1.8 × 10^4^ M^−1^ s^−1^ and a *k*_off_ rate of 1.2 × 10^−2^ s^−1^ (K_D_ value: 6.5 × 10^−7^ M). These results indicate that FasMab has a stronger affinity than typical anti-carbohydrate antibodies.

### Measurement of fucosylated AFP in serum using the fully automated analyzer

**Figure 7:**
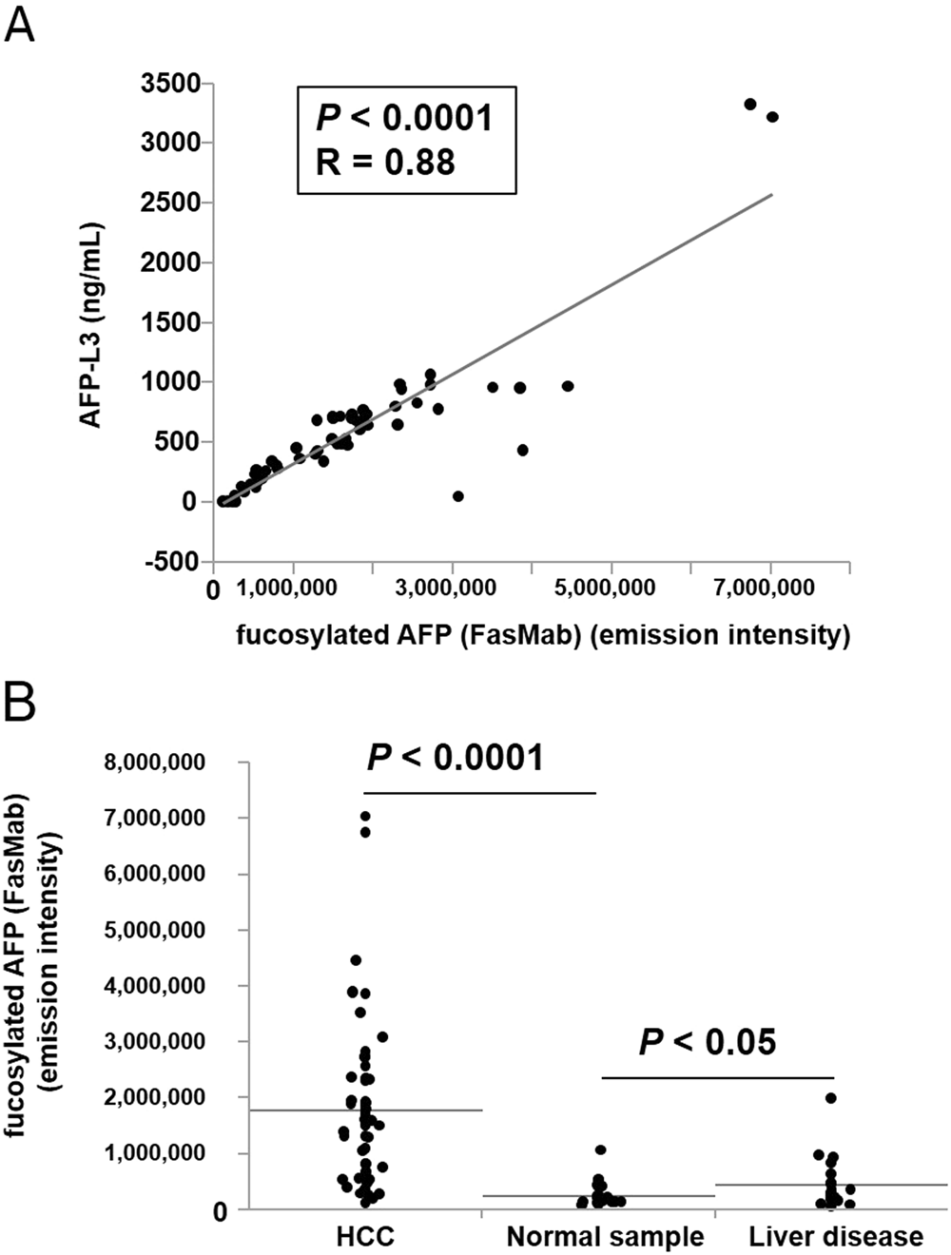
Establishment of a preliminary HISCL kit for measuring fucosylated AFP in the sera of HCC patients. (**A**) Graph showing the correlation between AFP-L3 concentrations (measured using µTAS WAKO AFP-L3) and fucosylated AFP levels, measured using a preliminary HISCL kit. (**B**) Fucosylated AFP levels from the sera of patients with HCC, normal patients, and patients with liver disease, were measured with a preliminary HISCL kit and FasMab.

In order to measure fucosylated AFP levels in sera, we used the HISCL-800, an automated chemiluminescent enzyme immunoassay system, and developed preliminary reagents for fucosylated AFP measurement with FasMab-coated magnetic particle, ALP-conjugated rabbit anti-AFP mAb in-house developed, and CDP-Star as a substrate. We determined that the limit of quantitation of the assay was 0.8 ng/mL, which was comparable sensitivity to that of AFP-L3 kit (0.3 ng/mL)^9^. Using this preliminary FasMab HISCL kit, we measured sera of 25 normal patients, 20 patients with benign liver diseases, and 55 patients with AFP-positive HCC. As shown in Fig. 7A, the levels of fucosylated AFP in HCC patient sera were strongly correlated with AFP-L3 values determined using μTAS WAKO with the exception of a few cases. Levels of fucosylated AFP were significantly increased in HCC patients compared with those in non-HCC patients (Fig. 7B), suggesting that levels of fucosylated AFP levels can be determined using HISCL, and may be very useful biomarkers for HCC.

## Discussion

In this study, we developed and characterized a novel glycan antibody, FasMab, which is specific for fucosylated AFP. Although conventional glycan antibodies recognize characteristic oligosaccharide modifications, FasMab did not react to α-1,6-fucosylated ALP and afucosylated AFP, but only to fucosylated AFP (Fig. 4A). In addition, FasMab did not react to fucosylated proteins other than AFP in the conditioned media of wild-type HepG2 cells (Fig. 4C). These results suggest that the FasMab epitopes include both fucosylated *N*-glycans and the AFP peptide. Our epitope mapping results indicate that the peptide epitope is within the TKVNFT amino acid sequence which includes Asn-232 as *N*-glycosylation site (Fig. 2). Since α-1,6-fucosylation is common glycoform observed in antibody-producing animals like mice and rabbits, α-1,6-fucosylated AFP itself has low immunogenicity. In order to overcome this difficulty in our study we used the characteristic AFP glycopeptide, with core fucose, for immunization and could obtain fucosylated AFP specific antibody. Although there is no previous example except for fucosylated AFP, the glycopeptide immunization method is thought to be applied to obtain other glycoprotein specific mAbs in principle. However, in the case of glcyoproteins with many *N*-glycans, the synthesized peptide design seems to be difficult.

The binding activities of FasMab to the two fucosylated AFP peptides tested were very similar; these AFP peptide sequences were the same except for the non-reducing termini of the *N*-glycans (Fig. 2). Since FasMab binds not only to AFP peptide with short *N*-glycan such as GlcNAc_2_ManFuc (Fig. 2) but also fucosylated AFP and AFP from wild-type HepG2 cells (Fig. 5) in which major *N*-glycan are highly elongated (branched and sialylated), it is thought that FasMab recognizes fucosylated *N*-glycan without effect from non-reducing termini of *N*-glycans and these results indicate that FasMab binds not only AFP modified with bi-antennary fucosylated *N*-glycans, but also AFP modified with branched fucosylated *N*-glycans unlike LCA lectin^29,30^ which was used in AFP-L3 measurement.

Since most anti-carbohydrate antibodies have low affinity for their antigens, we investigated whether the FasMab was specific for fucosylated AFP using SPR analysis^26–28^. The K_D_ value of Fab of FasMab to fucosylated AFP with SPR analysis was 6.5 × 10^−7^ M, which is higher than conventional glycan antibodies^28^; this affinity could be strong enough to detect fucosylated AFP in clinical samples.

The reactivity of FasMab in sandwich ELISA was very low under normal conditions (data not shown), although FasMab functioned for use in western blot analysis. However, we found that addition of 4 mM TCEP enhanced the specific binding of FasMab to fucosylated AFP (Fig. 5). These data suggest that denaturation of fucosylated AFP is required for FasMab to efficiently bind to glycan modifications. Therefore, reduction of the AFP three-dimensional structure is likely a major factor that enables FasMab to access its epitope.

We further tested the ability of FasMab to detect fucosylated AFP in the sera using an HISCL system. In the case of HCC samples, we found that fucosylated AFP levels highly correlated with AFP-L3 values (Fig. 7). However, several points were deviated from the correlation curves, and almost all of the outliers have higher fucosylated AFP levels and lower AFP-L3 value. One possibility is that fucosylated AFP-L3 interacted with other proteins and/or substances, which inhibit the binding to LCA lectin. Another possibility is that serum proteins denatured by reducing agent bound ALP-labeled detection antibody in a non-specific manner.

On the other hand, non-HCC samples (normal samples and liver disease samples) had significantly lower fucosylated AFP levels. Also in this case, however, a few samples showed high levels of fucosylated AFP, which could be due to a non-specific reaction.

To solve these problems and improve this preliminary system, further studies are planned to clarify the cause of these discrepancies. These future studies will determine whether the FasMab-HISCL system can be reliably used to measure fucosylated AFP levels in HCC patients.

We have investigated molecular mechanisms underlying by which AFP-L3 is specifically increased in sera of patients with HCC, but not benign liver diseases although expression of FUT8 in the liver is increased in chronic liver diseases such as chronic hepatitis and liver cirrhosis. We have speculated that fucosylated proteins in the liver are selectively secreted into apical side, bile structure and this system is broken in HCC due to loss of cellular polarity. FasMab could be a powerful tool to solve this question.

In conclusion, we have succeeded in the development of a novel anti-glycan antibody, FasMab, which recognizes fucosylated AFP and has promising potential for clinical use in the diagnosis of HCC.

## Supplementary information

Supplemental Information
