# Supplementary material for: Establishment and characterization of a fucosylated α-fetoprotein-specific monoclonal antibody: a potential application for clinical research
Source: Sci Rep. 2019 Aug 26;9:12359. doi: 10.1038/s41598-019-48821-x (PMC6710264; doi:10.1038/s41598-019-48821-x)
Supplement: Supplementary file 1 — Supplemental Information [file 41598_2019_48821_MOESM1_ESM.pdf]

Supplemental Information

Establishment and characterization of a fucosylated α-fetoprotein-specific monoclonal antibody: a potential application for clinical research

Yuriko Egashira[1], Masatoshi Suganuma[2], Yukiko Kataoka[1], Yukiko Higa[1], Nobuyuki Ide[3], Koichi Morishita[4], Yoshihiro Kamada[4], Jianguo Gu[5], Koji Fukagawa[1*], Eiji Miyoshi[4*]

[1]*Bio-Diagnostic Reagent Technology Center, Sysmex Corporation, Takatsukadai, Nishi-ku, Kobe, 651-2271, Japan;* [2]*Central Research Laboratory, Sysmex Corporation, Tonomachi, Kawasaki-ku, Kawasaki, Kanagawa, 210-0821, Japan;* [3]*Department of Technology Development, Sysmex Corporation, Takatsukadai, Nishi-ku, Kobe, 651-2271, Japan;* [4]*Department of Molecular Biochemistry & Clinical Investigation, Osaka University Graduate School of Medicine, Yamada-oka, Suita, 565-0871, Japan;* [5]*Division of Regulatory Glycobiology, Institute of Molecular Biomembrane and Glycobiology, Tohoku Medical and Pharmaceutical University, Komatsushima, Aobaku, Sendai 981-8558, Miyagi, Japan*

*Correspondence: Koji Fukagawa (Bio-Diagnostic Reagent Technology Center, Sysmex Corporation, Takatsukadai, Nishi-ku, Kobe, 651-2271, Japan, Tel: 81-78-991-1911, Fax: 81-78-992-3284, E-mail: Fukagawa.Koji@sysmex.co.jp), or Eiji Miyoshi (Department of Molecular Biochemistry & Clinical Investigation, Osaka University Graduate School of Medicine, 1-7 Yamada-oka, Suita, 565-0871, Japan, TEL & Fax: 81-6-6879-2590, E-mail: emiyoshi@sahs.med.osaka-u.ac.jp).

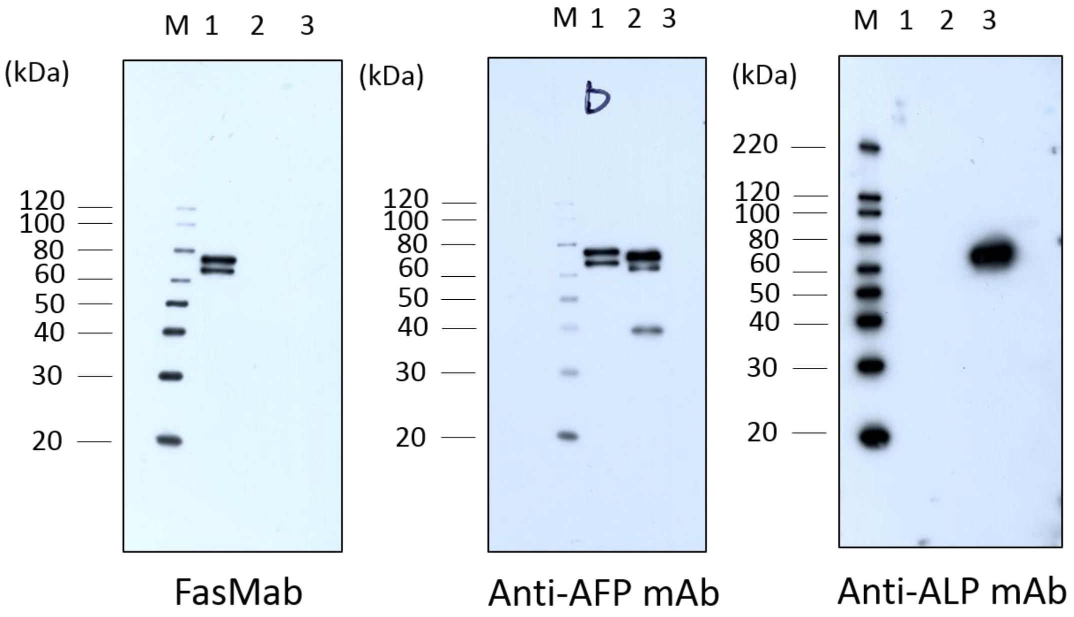

Supplementary Figure 1. Original images of western blot analyses for purified fucosylated AFP, afucosylated AFP and ALP.

Western blot analyses for purified fucosylated AFP, afucosylated AFP, and ALP. Lane M: marker (MagicMark XP Western Protein Standard; Thermo Fisher Scientific), Lane 1: fucosylated AFP, Lane 2: afucosylated AFP, Lane 3: ALP. Specific fluorescent bands were detected with X-ray Film RX-U (FUJIFILM, Tokyo, Japan).

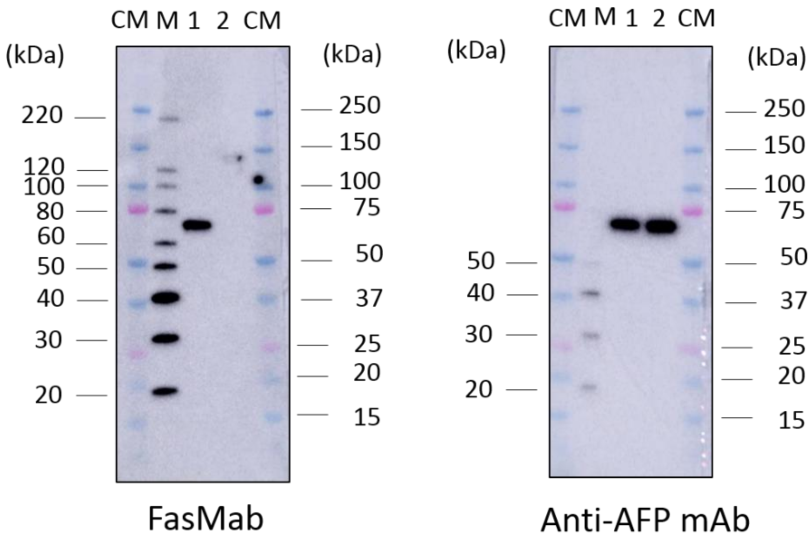

Supplementary Figure 2. Original images of western blot analyses for purified fucosylated AFP, afucosylated AFP and ALP.

Western blot analyses for AFP purified from conditioned media of wild-type and *FUT8*-deficient HepG2 cells. Lane CM: color marker (Precision Plus Protein Dual Color Standards; Bio-Rad), Lane M: marker (MagicMark XP Western Protein Standard; Thermo Fisher Scientific), Lane 1: AFP from wild-type HepG2 cells, Lane 2: AFP from *FUT8*-deficient HepG2 cells. Specific fluorescent bands were detected with an Amersham Imager 600 (GE Healthcare).

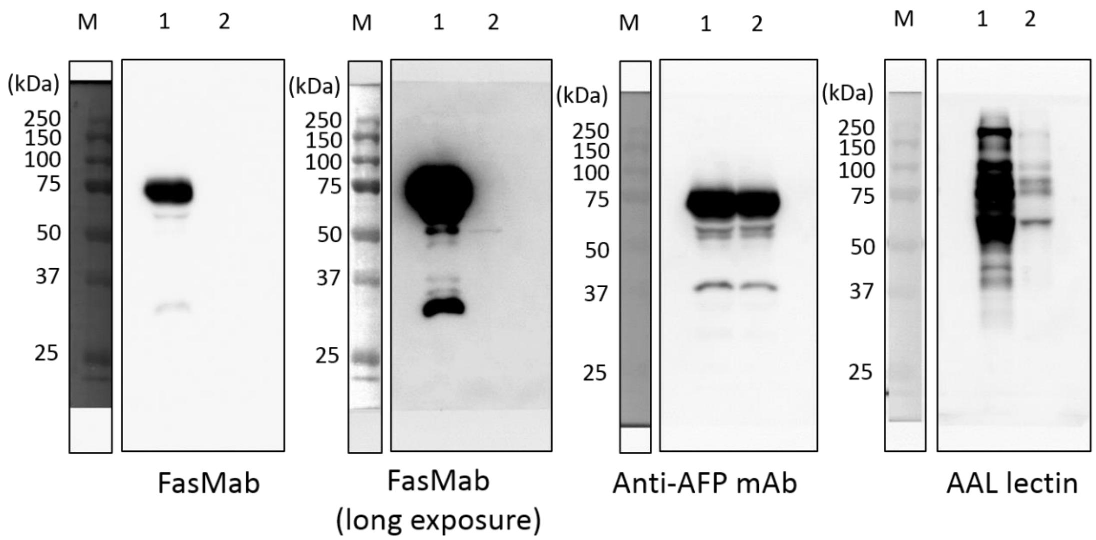

Supplementary Figure 3. Full-length images of western blot analyses of FasMab, anti-AFP mAb and AAL, using the conditioned media of wild-type and *FUT8*-deficient HepG2 cells.

Western blot and AAL blot analyses were performed, as described in Materials and Methods. 20 μg of condensed supernatant proteins were used in these experiments Lane 1: wild-type HepG2 cells, lane 2: *FUT8*-deficient HepG2 cells. Specific fluorescent bands were detected with an ImageQuant LAS 4000 (GE Healthcare).

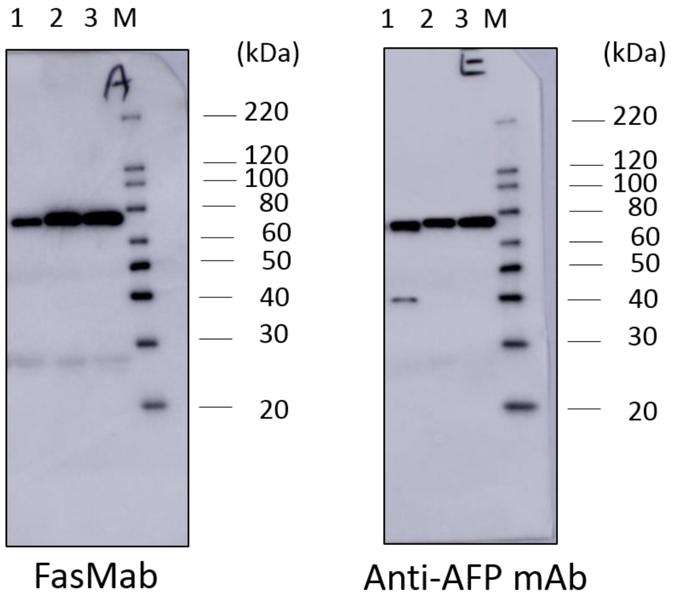

Supplementary Figure 4. Original images of western blot analyses for AFP following co-precipitation with an anti-AFP polyclonal antibody from human serum.

Western blot analyses for AFP following co-precipitation with an anti-AFP polyclonal antibody from human serum. Lane 1: sample 1, Lane 2: sample 2, Lane 3: sample 3, Lane M: marker (MagicMark XP Western Protein Standard; Thermo Fisher Scientific). Specific fluorescent bands were detected with an Amersham Imager 600 (GE Healthcare).
